# Prevalence and related factors of nephrolithiasis among medical staff in Qingdao, China: a retrospective cross-sectional study

**DOI:** 10.1186/s12882-024-03651-6

**Published:** 2024-07-01

**Authors:** Lei Guo, Lijun Liu, Ying Sun, Li Xue, Xingji Gong, Yue Wang, Wei Jiao, Haitao Niu

**Affiliations:** 1 Department of Urology, The Affiliated Hospital of Qingdao University, Qingdao, Shandong 266000 China; 2 Department of Neurology, The Affiliated Hospital of Qingdao University, Qingdao, Shandong 266000 China; 3 Health Management Center, The Affiliated Hospital of Qingdao University, Qingdao, Shandong 266000 China; 4 Department of Emergency, The Affiliated Hospital of Qingdao University, Qingdao, Shandong 266000 China; 5 Information Management Department, The Affiliated Hospital of Qingdao University, Qingdao, Shandong 266000 China

**Keywords:** Prevalence, Nephrolithiasis, Medical staff, Risk factors, Cross-sectional study

## Abstract

**Background:**

Certain occupations may predispose individuals to urolithiasis, a multi-factorial disease. The study aimed to evaluate the prevalence and related factors of nephrolithiasis in medical staff in Qingdao, China.

**Methods:**

Physical examination results of 5115 in-service medical staff aged 22–60 years old were retrospectively analyzed. Multivariable logistic regression analysis and stratified analyses by age and gender were applied to explore the related factors of nephrolithiasis in these medical staff.

**Results:**

The overall nephrolithiasis prevalence in medical staff in Qingdao, China was 4.65%. Doctors were more prone to nephrolithiasis than nurses (5.63% vs. 3.96%, *P* = 0.013) and the peak prevalence (6.69%) was observed in medical staff working in the emergency department (ED). Male gender (OR = 1.615, 95% CI = 1.123–2.323, *P* = 0.010), overweight or obesity (OR = 1.674, 95% CI = 1.266–2.214, *P* < 0.001), work seniority ≥ 10 years (OR = 2.489, 95%CI = 1.675–3.699, *P* < 0.001) and working in the ED (OR = 1.815, 95% CI = 1.202–2.742, *P* = 0.005) were independent predictors for nephrolithiasis in medical staff based on the results of multivariate logistic regression analysis. The associations between overweight or obesity and nephrolithiasis risk as well as between work seniority ≥ 10 years and nephrolithiasis risk in medical staff were independent of age or gender in stratified analysis.

**Conclusions:**

Nephrolithiasis prevalence in medical staff in Qingdao, China seemed not to be higher than that in the general population. Medical staff with work seniority ≥ 10 years and working in the ED should pay abundant attention to take measures to modify their nephrolithiasis risk.

## Background

Urolithiasis, namely urinary stone disease (USD), is one of the most common urological diseases with varied prevalence rates ranging from 1 to 20% depending on genetic, sociodemographic, geographic, climatic and lifestyle factors [1–3]. It may be asymptomatic or lead to symptoms necessitating intervention (e.g., flank pain, dysuria, hematuria), or even result in severe consequences such as pyonephrosis and chronic renal failure [4, 5]. Various factors contribute to stone formation, including urinary infection and obstruction, genetic predisposition, climate, gender, age, obesity, weight gain, diet, limited fluid intake, certain drugs and metabolic disorders [6–8]. Certain occupations may also predispose individuals to urolithiasis. Glass plant workers, cooks, engineering room personnel and steel workers have been reported to be at a higher risk of urolithiasis due to dehydration associated with high temperature exposure and perspiration [9–11].

Medical staff are engaged in busy and stressful work, which may affect their dietary habits, fluid intake, psychological condition, sleep, and so on. Stress and poor sleep quality have also been demonstrated to be associated with the risk of kidney stones [12, 13]. It seems that medical staff are more susceptible to urolithiasis than the general population, but there has been limited data about this topic. The present study aimed to evaluate the prevalence and related factors of nephrolithiasis among medical staff in Qingdao, China, which have not been investigated to date.

## Methods

### Study design and population

The study was a retrospective, cross-sectional and single-center study. The latest physical examination for employees in the Affiliated Hospital of Qingdao University was conducted between January and May 2023 and the results were retrospectively collected. The Ethics Committee of this hospital approved this study and waived the informed consents given the retrospective nature of the study.

Data of employees working in non-healthcare positions (administrative staff, logistics and laboratory staff, medical record practitioners, social workers, etc.) were abandoned because their jobs distinctly differ from those of medical staff. Retired medical staff were also excluded from this study. Physical examination results of in-service medical staff (doctors, nurses, pharmacists, technicians, dieticians and rehabilitation therapists) were manually evaluated and those without body mass index (BMI), blood pressure (BP) values or adequate blood test results for judgment of comorbidities (hypertension, diabetes mellitus, hyperuricemia and overweight or obesity) or without renal ultrasound, kidney-ureter-bladder plain film (KUB) or abdominal computed tomography (CT) results for determination of nephrolithiasis were further excluded.

### Data collection

The following data of enrolled medical staff were systematically and retrospectively collected from physical examination records: age, gender, occupations, working department, history of diseases (nephrolithiasis, hypertension, diabetes mellitus and hyperuricemia), smoking status, BMI, levels of blood glucose and uric acid and results of renal ultrasound, KUB or abdominal CT. In addition, we collected work arrangement (whether they were on night shifts) and work seniority (< 10 years or ≥ 10 years) of each subject from the corresponding departments.

Subjects with nephrolithiasis included those who had a history of renal calculi regardless whether they received treatment and those who were confirmed to have renal calculi under renal ultrasound, KUB or abdominal CT regardless of whether they had symptoms. Echogenic foci (with or without acoustic shadowing) under renal ultrasound or high-density shadows under KUB or abdominal CT in the renal pelvis or calices were diagnosed as renal stones [14].

Identification of hypertension, diabetes mellitus and hyperuricemia were based on past medical history and diagnostic criteria in corresponding guidelines [15–17]. Overweight or obesity was defined as BMI 24 kg/m^2^ or greater according to the cutoff proposed for Chinese adults and the corresponding Chinese guideline [18, 19].

### Statistical analysis

Data were processed through Microsoft Excel 2013 and analyzed through IBM SPSS software version 25.0. A two-tailed *P* value < 0.05 was considered statistically significant and the Bonferroni correction was used to adjust the level of significance for multiple comparisons. Ages of the enrolled subjects in this study didn’t comply with the normal distribution (*P* < 0.05 in Kolmogorov–Smirnov test), therefore median with interquartile range (IQR) was used to describe this variable and Mann–Whitney U test was adopted to estimate the intergroup discrepancies. Categorical variables were summarized as frequencies and percentages and were compared by the Pearson’s chi-square test.

To explore any associations between potential risk factors and nephrolithiasis in medical staff, univariate logistic regression analysis was performed and variables with a *P* value of < 0.10 were subsequently entered into a multivariate logistic regression analysis with the results presented as odds ratios (ORs) and 95% confidence intervals (CIs). Additionally, a sensitivity analysis was conducted to assess the stability of results, in which ORs and 95%CIs were recalculated with all variables entered into the multivariate logistic regression analysis. Stratified analysis was further carried out to examine the associations between potential risk factors with nephrolithiasis risk, separated by age and gender, respectively.

## Results

### Characteristics of the enrolled medical staff

**Fig. 1:**
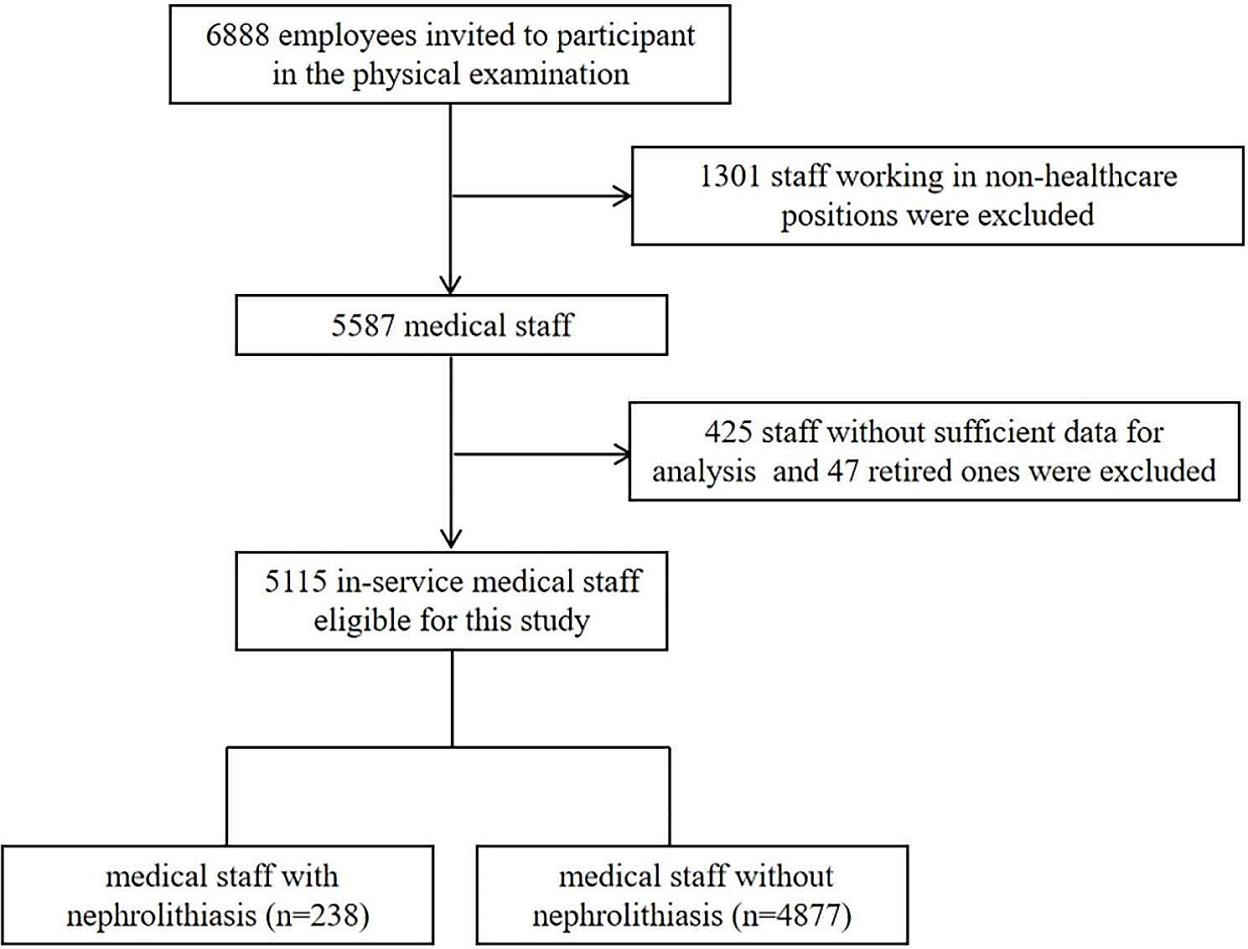
The flow chart of the study population inclusion

A total of 6888 employees, including 1301 staff working in non-healthcare positions, were initially invited to participate in the physical examination. Among the 5587 medical staff, 425 ones without sufficient data for analysis and 47 ones who have retired were excluded, leaving 5115 in-service medical staff eligible for analysis. The flow chart of the study population inclusion is detailed in Fig. 1.

There were 1025 men and 4090 (79.96%) women, aged 22–60 (median 34) years old. 238 medical staff were identified to suffer from nephrolithiasis, accounting for 4.65% of the overall study population. Prevalence rates of smoking, hypertension, diabetes mellitus, hyperuricemia and overweight or obesity were 7.90%, 6.61%, 1.17%, 9.05% and 32.75%, respectively. In terms of work-related factors, 72.98% of them were on night shifts and 41.15% of them were with work seniority of greater than 10 years. The proportions of doctors, nurses, other medical staff (pharmacists, technicians, dieticians and rehabilitation therapists), emergency department (ED) medical staff and operating room medical staff were 26.39%, 60.24%, 13.37%, 8.88% and 17.95%, respectively. Characteristics of the enrolled medical staff are presented in Table 1.

**Table 1:** Characteristics of the enrolled medical staff and comparisons between those with and without nephrolithiasis

	Overall (*n* = 5115)	Nephrolithiasis		*P* value
		Yes (*n* = 238)	No (*n* = 4877)	
**Demographics**				
Age (year), median (IQR)	34 (30–39)	36 (32–42)	34 (29–39)	***< 0.001***
Female, *n* (%)	4090 (79.96)	158 (66.39)	3932 (80.62)	***< 0.001***
**Current smoking,** ***n*** **(%)**	404 (7.90)	24 (10.08)	380 (7.79)	0.200
**Comorbidities**				
Hypertension, *n* (%)	338 (6.61)	32 (13.45)	306 (6.27)	***< 0.001***
Diabetes mellitus, *n* (%)	60 (1.17)	5 (2.10)	55 (1.13)	0.292
Hyperuricemia, *n* (%)	463 (9.05)	32 (13.45)	431 (8.84)	***0.016***
Overweight or obesity, *n* (%)	1675 (32.75)	117 (49.16)	1558 (31.95)	***< 0.001***
**Night shifts,** ***n*** **(%)**	3733 (72.98)	165 (69.33)	3568 (73.16)	0.194
**Work seniority,** *n* **(%)**				***< 0.001***
< 10 years	3010 (58.85)	92 (38.66)	2918 (59.83)	
≥ 10 years	2105 (41.15)	146 (61.34)	1959 (40.17)	
**Working positions**				***0.015***
Doctors	1350 (26.39)	76 (31.93)	1274 (26.12)	
Nurses	3081 (60.24)	122 (51.26)	2959 (60.67)	
Other medical staff	684 (13.37)	40 (16.81)	644 (13.20)	
**Working in the ED**	454 (8.88)	30 (12.61)	424 (8.69)	***0.038***
**Working in the operating room**	918 (17.95)	49 (20.59)	869 (17.82)	0.277

IQR: interquartile range; ED: emergency department

The *P* values indicated in bold and italic are statistically significant

Given disparities in workload and stress levels, comparisons of nephrolithiasis prevalence were conducted among doctors, nurses and other medical staff, between ED and non-ED medical staff as well as between operating room and non-operating room medical staff, respectively. Doctors were more prone to nephrolithiasis than nurses (5.63% vs. 3.96%, *P* = 0.013 in Bonferroni-corrected post hoc analysis). In contrast, the nephrolithiasis prevalence rate in other medical staff (5.85%) was not significantly different from those in doctors (*P* = 0.841) and in nurses (*P* = 0.028) after Bonferroni correction in Pearson’s chi-square test. Moreover, nephrolithiasis was apparently more prevalent in ED medical staff (6.61%) than their non-ED colleagues (4.46%) (*P* = 0.038). However, the difference of nephrolithiasis prevalence was not statistically significant between the operating room (5.34%) and non-operating room employees (4.50%) (*P* = 0.277).

### Related factors of nephrolithiasis in medical staff

Comparisons between medical staff with and without nephrolithiasis were shown in Table 1.

Univariate analysis (Mann–Whitney U test and Pearson’s chi-square test) suggested that the intergroup differences in age (*P* < 0.001), gender (*P* < 0.001), hypertension (*P* < 0.001), hyperuricemia (*P* = 0.016), overweight or obesity (*P* < 0.001), working positions (*P* = 0.015) and the proportions of those with work seniority ≥ 10 years (*P* < 0.001) and ED staff (*P* = 0.038) were statistically significant.

The results of univariate logistic regression analysis showed that age (OR = 1.038, 95%CI = 1.023–1.053, *P* < 0.001), male gender (OR = 2.107, 95%CI = 1.595–2.782, *P* < 0.001), hypertension (OR = 2.320, 95%CI = 1.571–3.427, *P* < 0.001), hyperuricemia (OR = 1.602, 95%CI = 1.090–2.356, *P* = 0.016), overweight or obesity (OR = 2.060, 95%CI = 1.586–2.675, *P* < 0.001) work seniority ≥ 10 years (OR = 2.364, 95%CI = 1.810–3.088, *P* < 0.001) and working in the ED (OR = 1.515, 95%CI = 1.020–2.250, *P* = 0.040) were risk factors for nephrolithiasis in medical staff (Table 2). In contrast to working in a doctor position, working in a nurse position was a protective factor for kidney stone formation (OR = 0.691, 95%CI = 0.515–0.927, *P* = 0.014) (Table 2). Then the above factors were analyzed by enter method of multivariate logistic regression analysis, revealing that male gender (OR = 1.615, 95%CI = 1.123–2.323, *P* = 0.010), overweight or obesity (OR = 1.674, 95%CI = 11.266–2.214, *P* < 0.001), work seniority ≥ 10 years (OR = 2.489, 95%CI = 1.675–3.699, *P* < 0.001) and working in the ED (OR = 1.815, 95%CI = 1.202–2.742, *P* = 0.005) remained independent risk factors for nephrolithiasis in medical staff (Table 2). Based on these results, we included more covariates including smoking, diabetes mellitus, night shifts and working in the operating-room to perform further sensitivity analysis, and the results were consistent with those of the multivariate logistic regression analysis (male gender: OR = 1.644, 95%CI = 1.122–2.411, *P* = 0.011; overweight or obesity: OR = 1.667, 95%CI = 1.261–2.205, *P* < 0.001; work seniority ≥ 10 years: OR = 2.408, 95%CI = 1.615–3.591, *P* < 0.001; working in the ED: OR = 1.797, 95%CI = 1.176–2.746, *P* = 0.007), indicating result stability (Table 2).

**Table 2:** Results of logistic regression analysis of the influencing factors of nephrolithiasis in medical staff

	Unadjusted		Adjusted		Sensitivity analysis	
	OR (95%CI)	*P* value	OR (95%CI)	*P* value	OR (95%CI)	*P* value
**Age**	1.038 (1.023–1.053)	***< 0.001***	0.989 (0.965–1.014)	0.392	0.996 (0.969–1.024)	0.770
**Gender**						
Female	Reference		Reference		Reference	
Male	2.107 (1.595–2.782)	***< 0.001***	1.615 (1.123–2.323)	***0.010***	1.644 (1.122–2.411)	***0.011***
**Current smoking**	1.327 (0.859–2.050)	0.202	-	-	0.884 (0.551–1.418)	0.610
**Hypertension**	2.320 (1.571–3.427)	***< 0.001***	1.247 (0.805–1.931)	0.323	1.268 (0.815–1.972)	0.292
**Diabetes mellitus**	1.881 (0.746–4.744)	0.180	-	-	1.038 (0.401–2.690)	0.938
**Hyperuricemia**	1.602 (1.090–2.356)	***0.016***	0.958 (0.620–1.482)	0.848	0.956 (0.618–1.479)	0.840
**Overweight or obesity**	2.060 (1.586–2.675)	***< 0.001***	1.674 (1.266–2.214)	***< 0.001***	1.667 (1.261–2.205)	***< 0.001***
**Night shifts**	0.829 (0.625-1.100)	0.194	-	-	1.223 (0.852–1.756)	0.275
**Work seniority**						
<10 years	Reference		Reference		Reference	
≥10 years	2.364 (1.810–3.088)	***< 0.001***	2.489 (1.675–3.699)	***< 0.001***	2.408 (1.615–3.591)	***< 0.001***
**Working positions**		***0.015***		0.647		0.421
Doctors	Reference		Reference		Reference	
Nurses	0.691 (0.515–0.927)	***0.014***	1.019 (0.711–1.460)	0.919	1.051 (0.721–1.533)	0.796
Other medical staff	1.041 (0.702–1.545)	0.841	1.199 (0.799–1.798)	0.381	1.345 (0.846–2.139)	0.211
**Working in the ED**	1.515 (1.020–2.250)	***0.040***	1.815 (1.202–2.742)	***0.005***	1.797 (1.176–2.746)	***0.007***
**Working in the operating room**	1.196 (0.866–1.651)	0.277	-	-	1.085 (0.742–1.587)	0.673

OR: odds ratio; CI: confidence interval; ED: emergency department

The *P* values indicated in bold and italic are statistically significant

### Stratified analysis

We further detected the associations between potential risk factors and nephrolithiasis in medical staff, separated by age and gender, respectively.

When stratifying our analysis by age (Table 3), we divided all the participants into two subgroups based on the median age. Among the subjects ≤ 34 years old, male gender (OR = 2.243, 95%CI = 1.270–3.961, *P* = 0.005), overweight or obesity (OR = 1.827, 95%CI = 1.175–2.841, *P* = 0.007) and work seniority ≥ 10 years (OR = 16.387, 95%CI = 8.859–30.311, *P* < 0.001) were independently related to a higher risk of nephrolithiasis, but working in the ED was not an independent predictor (OR = 1.634, 95%CI = 0.919–2.908, *P* = 0.095). Among the subjects > 34 years old, overweight or obesity (OR = 1.638, 95%CI = 1.132–2.371, *P* = 0.009), work seniority ≥ 10 years (OR = 2.223, 95%CI = 1.179–4.193, *P* = 0.014) and working in the ED (OR = 2.315, 95%CI = 1.272–4.215, *P* = 0.006) were independently related to a higher risk of nephrolithiasis, but male gender was not an independent predictor (OR = 1.206, 95%CI = 0.756–1.924, *P* = 0.432). These results indicated that the associations between overweight or obesity and nephrolithiasis risk as well as between working seniority ≥ 10 years and nephrolithiasis risk were not affected by age in medical staff. In contrast, the associations between male gender and nephrolithiasis risk as well as working in the ED and nephrolithiasis risk were influenced by age.

**Table 3:** Stratified analysis for the associations between influencing factors and nephrolithiasis risk by age

	Age, OR (95%CI)			
	≤ 34 (*n* = 2683)	*P* value	> 34 (*n* = 2432)	*P* value
**Gender**				
Female	Reference		Reference	
Male	2.243 (1.270–3.961)	***0.005***	1.206 (0.756–1.924)	0.432
**Hypertension**	0.736 (0.215–2.525)	0.626	1.514 (0.945–2.425)	0.084
**Hyperuricemia**	1.168 (0.591–2.308)	0.655	0.831 (0.464–1.487)	0.532
**Overweight or obesity**	1.827 (1.175–2.841)	***0.007***	1.638 (1.132–2.371)	***0.009***
**Work seniority**				
<10 years	Reference		Reference	
≥10 years	16.387 (8.859–30.311)	***< 0.001***	2.223 (1.179–4.193)	***0.014***
**Working positions**		0.446		0.111
Doctors	Reference		Reference	
Nurses	0.796 (0.448–1.417)	0.439	0.825 (0.525–1.295)	0.403
Other medical staff	0.583 (0.250–1.357)	0.211	1.408 (0.887–2.236)	0.147
**Working in the ED**	1.634 (0.919–2.908)	0.095	2.315 (1.272–4.215)	***0.006***

OR: odds ratio; CI: confidence interval; ED: emergency department

The *P* values indicated in bold and italic are statistically significant

Gender, hypertension, hyperuricemia, overweight or obesity, work seniority, working positions and working in the ED were adjusted in the multivariate logistic regression analysis

Stratified analysis by gender (Table 4) showed that overweight or obesity (female: OR = 1.517, 95%CI = 1.083–2.124, *P* = 0.015; male: OR = 2.086, 95%CI = 1.238–3.514, *P* = 0.006) and work seniority ≥ 10 years (female: OR = 2.397, 95%CI = 1.465–3.922, *P* = 0.001; male: OR = 2.462, 95%CI = 1.230–4.930, *P* = 0.011) were independently associated with a higher risk of nephrolithiasis in both female and male medical staff. Working in the ED (OR = 2.202, 95%CI = 1.372–3.533, *P* = 0.001) independently contributed to kidney stone formation only in female. In other words, the associations between overweight or obesity and nephrolithiasis risk as well as between working seniority ≥ 10 years and nephrolithiasis risk in medical staff were not affected by gender. Nevertheless, the association between working in the ED and nephrolithiasis risk were influenced by gender.

**Table 4:** Stratified analysis for the associations between influencing factors and nephrolithiasis risk by gender

	Age, OR (95%CI)			
	Female (*n* = 4090)	*P* value	Male (*n* = 1025)	*P* value
**Age**	0.998 (0.965–1.031)	0.889	0.977 (0.941–1.015)	0.241
**Hypertension**	1.731 (0.917–3.265)	0.090	0.965 (0.534–1.742)	0.906
**Hyperuricemia**	1.018 (0.402–2.579)	0.970	0.924 (0.565–1.511)	0.752
**Overweight or obesity**	1.517 (1.083–2.124)	***0.015***	2.086 (1.238–3.514)	***0.006***
**Work seniority**				
<10 years	Reference		Reference	
≥10 years	2.397 (1.465–3.922)	***0.001***	2.462 (1.230–4.930)	***0.011***
**Working positions**		0.862		0.554
Doctors	Reference		Reference	
Nurses	1.108 (0.712–1.724)	0.649	0.741 (0.348–1.577)	0.437
Other medical staff	1.166 (0.636–2.137)	0.619	1.159 (0.666–2.019)	0.601
**Working in the ED**	2.202 (1.372–3.533)	***0.001***	1.164 (0.500-2.709)	0.725

OR: odds ratio; CI: confidence interval; ED: emergency department

The *P* values indicated in bold and italic are statistically significant. Age, hypertension, hyperuricemia, overweight or obesity, work seniority, working positions and working in the ED were adjusted in the multivariate logistic regression analysis

## Discussion

As a multi-factorial disease, urolithiasis prevalence varies greatly across the world and exhibits a distinctive geographical distribution with the so-called ‘global stone belt’ running across the low- and mid-latitude regions [20]. In China, the average urolithiasis prevalence in adults was 6.5% in 2015 according to Zeng’s cross-sectional survey [21]. Again, extremely inhomogeneous spatial distribution was observed and the prevalence was markedly higher in South China (11.6%) than in East China (4.8%) [21, 22]. Qingdao is a city in Shandong province, located on the east coast of China. Although no data is available with respect to the prevalence of urolithiasis in Shandong province so far, it is lower than 5% based on Yang’s study [22]. The study is the first to quantify nephrolithiasis prevalence in medical staff from Qingdao and revealed a prevalence rate of 4.65%, which seemed not to be higher than that in the general population of Shandong province.

Our findings were similar to those of a previous study from Mayo Clinic, which also found that urolithiasis prevalence in employees of their institution (10.9%) was comparable to those reported in the United States population [23]. Interestingly, Chen and co-authors discovered that physicians even had a lower urolithiasis risk than did the general population [24]. In view of these results, we suppose that although medical staff are exposed to a variety of risk factors contributing to urolithiasis as mentioned in the background part, extensive medical knowledge and higher disease awareness may promote them to take action to combat with the high risk of stone formation. Notably, the restricted age range (22–60 years old) and higher female proportion (79.96%) of participants in this study may influence the comparison of nephrolithiasis prevalence between medical staff and the general population, because urolithiasis prevalence increased with age, peaked in people aged 65–74 (8.2%) and ≥ 75 (8.5%) years old and affected men 1.33 times more frequently than women in China [21].

Potential risk factors for nephrolithiasis in medical staff explored in our study could be mainly classified into demographic factors, lifestyle factors, comorbidities and work-related factors. With regard to demographic, lifestyle factors and comorbidities, the links between age, male gender, hypertension, hyperuricemia, obesity and USD have been documented by numerous studies [21, 25, 26]. Male gender and overweight or obesity have been further confirmed to be independently associated with nephrolithiasis risk in medical staff in Qingdao. Up to now, the part of smoking in urolithiasis is controversial. The observational study by Huang et al. revealed that current smoking and high serum cotinine concentrations may be associated with an increased risk of kidney stones [27]. On the contrary, no credible evidence was identified that cigarette smoking influenced the occurrence and recurrence of urolithiasis in Detsyk’s study [28]. We didn’t identify the link between smoking and nephrolithiasis in medical staff from Qingdao either.

We paid special attention to the roles of work-related factors in USD in medical staff, including night shifts, work seniority, working positions, working in the ED and the operating room. Work seniority ≥ 10 years was verified to be an independent predictor for nephrolithiasis in medical staff and the association was not influenced by age or gender, probably because it means longer exposure to work-related risk factors. The highest nephrolithiasis prevalence was observed in ED medical staff and working in the ED was also revealed to be an independent predictor for nephrolithiasis risk in medical staff, but the relationships may be influenced by age and gender. Restricted fluid intake due to busy work was one of the factors. Moreover, ED medical staff face more critically ill patients, more rescues and more deaths, meaning a heavier workload and a greater deal of occupational stressors. Stress was identified to be a risk factor for urolithiasis and the plausible mechanisms involved production of hypertonic urine secondary to stress-induced secretion of vasopressin, elevated serum calcium levels secondary to stress-induced adrenocorticotropic hormone (ACTH), increased uric acid and inorganic phosphorus concentrations, reduced potassium and magnesium levels and so on [12, 29, 30]. These results remind us that medical staff with work seniority ≥ 10 years and working in the ED should pay abundant attention to take measures to modify their nephrolithiasis risk.

The correlation between night shift work and health is worth paying attention to, especially in medical staff. Night shift work results in a range of consequences, including sleep deprivation, sleep disturbance and daytime dysfunction, which have been indicated to play roles in urolithiasis [13]. Furthermore, an altered circadian clock experienced by shift workers and disrupted rhythm regularity due to incorrect exposure to bright light at night may also accelerate the development of kidney stones [31]. Nevertheless, the relevance of night shift work in nephrolithiasis was not validated in our cohort. Consistent with Chen’s findings [24], we uncovered that kidney stones were more common in doctors than in nurses and nurses had a lower nephrolithiasis risk according to the univariate analysis. However, working positions didn’t independently correlated with nephrolithiasis and they may co-act with other risk factors. In Linder’s study, working in the operating room was established to independently increase urolithiasis risk in health care professionals, especially in physicians [23]. Insufficient fluid intake owing to a busy operative schedule and higher stress levels associated with high work load, surgical complications and long working hours were proposed to be potential explanations [23]. However, the nephrolithiasis prevalence didn’t differ between operating room medical staff and their non-operating room colleagues in our study. Further studies concerning the effects of more work-related factors in multi-center and larger-scale studies are warranted.

Sufficient fluid intake has been consistently confirmed to prevent USD [32]. Restricted fluid intake due to busy work is common in medical staff and may contribute to the development of kidney stones. It is a pity that we didn’t have enough data about fluid intake of the participants for analysis in this study. Besides, we couldn’t assess the influences of other factors due to insufficient information, such as family history, lipid levels, other lifestyle factors (dietary habits, physical activity and exercise, drinking, etc.), nor could we establish the causalities between identified risk factors and nephrolithiasis due to the retrospective nature of the study. The present study had several other limitations. Firstly, as a single-center study, the sample size is limited and the results may not be generalized to medical staff in other areas. Secondly, locations, numbers and sizes of stones were not assayed and we didn’t discriminate between symptomatic and asymptomatic individuals. Thirdly, the absence of data from a control group with matched demographics made it impossible to directly compare the nephrolithiasis prevalence rates between medical staff and the general population in Qingdao. Finally, potential bias and inaccuracies brought by self- reported data, such as BMI and smoking in the study, may influence the reliability of the results.

Despite these limitations, the findings of the study provide new information for work-related risk factors associated with kidney stone formation in medical staff. Modification of the etiologies and risk factors is supposed to be the most effective way to prevent USD [32]. Except for the generally recommended protective strategies (adequate fluid intake, a healthy lifestyle [physical activity and exercise, avoidance of cigarette smoking and secondhand smoke, etc.], dietary management and caffeine consumption, etc.) [32], keeping a healthy pace of life involving regular schedules and relieving stress is also urgently required for nephrolithiasis prevention for this special occupational group.

## Conclusions

Our study demonstrated that nephrolithiasis prevalence in medical staff from Qingdao was 4.65%, which seemed not to be higher than that in the general population in Shandong province. Nephrolithiasis prevalence was higher in doctors than in nurses and the peak prevalence was observed in ED medical staff. As for work-related factors, work seniority ≥ 10 years and working in the ED were independently associated with nephrolithiasis susceptibility in medical staff from Qingdao. Further multi-center and larger-scale studies are warranted to ascertain nephrolithiasis prevalence and risk factors in medical staff.

## Data Availability

The datasets used and/or analyzed in the current study are available from the corresponding authors on reasonable request.

## Abbreviations

USD: urinary stone disease
BMI: body mass index
BP: blood pressure
KUB: kidney-ureter-bladder plain film
CT: computed tomography
IQR: interquartile range
OR: odds ratio
CI: confidence interval
ED: emergency department
ACTH: adrenocorticotropic hormone
